# Variation in racial/ethnic disparities in COVID-19 mortality by age in the United States: A cross-sectional study

**DOI:** 10.1371/journal.pmed.1003402

**Published:** 2020-10-20

**Authors:** Mary T. Bassett, Jarvis T. Chen, Nancy Krieger

**Affiliations:** 1 Harvard T.H. Chan School of Public Health, Boston, Massachusetts, United States of America; 2 Francois-Xavier Bagnoud Center for Health and Human Rights, Harvard University, Boston, Massachusetts, United States of America; Massachusetts General Hospital, UNITED STATES

## Abstract

**Background:**

In the United States, non-Hispanic Black (NHB), Hispanic, and non-Hispanic American Indian/Alaska Native (NHAIAN) populations experience excess COVID-19 mortality, compared to the non-Hispanic White (NHW) population, but racial/ethnic differences in age at death are not known. The release of national COVID-19 death data by racial/ethnic group now permits analysis of age-specific mortality rates for these groups and the non-Hispanic Asian or Pacific Islander (NHAPI) population. Our objectives were to examine variation in age-specific COVID-19 mortality rates by racial/ethnicity and to calculate the impact of this mortality using years of potential life lost (YPLL).

**Methods and findings:**

This cross-sectional study used the recently publicly available data on US COVID-19 deaths with reported race/ethnicity, for the time period February 1, 2020, to July 22, 2020. Population data were drawn from the US Census. As of July 22, 2020, the number of COVID-19 deaths equaled 68,377 for NHW, 29,476 for NHB, 23,256 for Hispanic, 1,143 for NHAIAN, and 6,468 for NHAPI populations; the corresponding population sizes were 186.4 million, 40.6 million, 2.6 million, 19.5 million, and 57.7 million. Age-standardized rate ratios relative to NHW were 3.6 (95% CI 3.5, 3.8; *p <* 0.001) for NHB, 2.8 (95% CI 2.7, 3.0; *p <* 0.001) for Hispanic, 2.2 (95% CI 1.8, 2.6; *p <* 0.001) for NHAIAN, and 1.6 (95% CI 1.4, 1.7; *p <* 0.001) for NHAP populations. By contrast, NHB rate ratios relative to NHW were 7.1 (95% CI 5.8, 8.7; *p <* 0.001) for persons aged 25–34 years, 9.0 (95% CI 7.9, 10.2; *p <* 0.001) for persons aged 35–44 years, and 7.4 (95% CI 6.9, 7.9; *p <* 0.001) for persons aged 45–54 years. Even at older ages, NHB rate ratios were between 2.0 and 5.7. Similarly, rate ratios for the Hispanic versus NHW population were 7.0 (95% CI 5.8, 8.7; *p <* 0.001), 8.8 (95% CI 7.8, 9.9; *p <* 0.001), and 7.0 (95% CI 6.6, 7.5; *p <* 0.001) for the corresponding age strata above, with remaining rate ratios ranging from 1.4 to 5.0. Rate ratios for NHAIAN were similarly high through age 74 years. Among NHAPI persons, rate ratios ranged from 2.0 to 2.8 for persons aged 25–74 years and were 1.6 and 1.2 for persons aged 75–84 and 85+ years, respectively. As a consequence, more YPLL before age 65 were experienced by the NHB and Hispanic populations than the NHW population—despite the fact that the NHW population is larger—with a ratio of 4.6:1 and 3.2:1, respectively, for NHB and Hispanic persons. Study limitations include likely lag time in receipt of completed death certificates received by the Centers for Disease Control and Prevention for transmission to NCHS, with consequent lag in capturing the total number of deaths compared to data reported on state dashboards.

**Conclusions:**

In this study, we observed racial variation in age-specific mortality rates not fully captured with examination of age-standardized rates alone. These findings suggest the importance of examining age-specific mortality rates and underscores how age standardization can obscure extreme variations within age strata. To avoid overlooking such variation, data that permit age-specific analyses should be routinely publicly available.

## Abstract 2

Mary Bassett and colleagues anzlyze US data on COVID-19 deaths to determine if, in addition to dying at higher rates, racial and ethnic minorities are also dying at younger ages.

## Author summary

**Why was this study done?:**

- Data from media coverage and local health department reports suggest that, as compared to the non-Hispanic White population, COVID-19 mortality rates are substantially higher among non-Hispanic Black, Hispanic, and non-Hispanic American Indian or Alaska Native populations.
- But no national data have been available to determine whether, in addition to dying at higher rates, these groups also die at younger ages.

**What did the researchers do and find?:**

- Using recently released national data on COVID-19 deaths by racial/ethnic group and age, along with US Census population data, we explored variation in mortality risk by calculating age-specific mortality measures in the above groups as well as in the non-Hispanic Asian or Pacific Islander population, the 5 census-defined groups for which data are available.
- We found that for all groups, as compared to the non-Hispanic White population, there were excess COVID-19 deaths across all ages. Although for all racial/ethnic groups, most deaths occurred at older ages, there was also striking loss of life at younger ages, before age 65, among people of color.
- More years of life were lost before 65 years among the non-Hispanic Black and Hispanic populations, despite the smaller size of these groups, than among the non-Hispanic White population. Scrutinizing age-specific mortality rates, we found that for young adults into midlife, comparatively, the non-Hispanic Black, Hispanic, and non-Hispanic American Indian or Alaska Native populations had a much higher risk of death from COVID-19 than the non-Hispanic White population.

**What do these findings mean?:**

- These data support the conclusion that US populations of color die of COVID-19 at younger ages as well as at higher rates than the non-Hispanic White population.
- Enhancing protection from infection, including assurance of workplace protections, may be relevant to prevention of COVID-19 in working-age adults.

## Introduction

The first death due to COVID-19 in the United States was reported on February 29, 2020. In late March, media reports brought to national attention the disproportionate number of COVID-19 cases and deaths occurring among the US Black and Latino populations [1]. Typically, these reports compared the proportion of cases and deaths by reported race/ethnicity to the racial/ethnic composition of the population. For example, a news report noted on March 27 that all (100%) of 8 deaths in Milwaukee were among Black Americans, who comprised 38% of the city’s population; in all of Wisconsin, only 15 deaths statewide had occurred [2]. Such reports came from state and local jurisdictions. At the time, the Centers for Disease Control and Prevention (CDC) made COVID-19 data publicly available only by age and sex, prompting many calls to release racial/ethnic data [3]. On April 6, New York City first released both crude and age-adjusted COVID-19 mortality rates, permitting some insight into the impact of population age structure and age at death on race/ethnicity-specific mortality rates [4]. Estimates of US national racial/ethnic mortality rate disparities, produced in mid-May, used indirect age standardization to compare rates, to address concerns about differences in population age structures [5]. Also suggesting that information on age-specific risks could be important, marked racial/ethnic inequities in premature morbidity and mortality are well documented for numerous health outcomes, reflecting inequities in working and living conditions [6–8]. Journalists additionally have become a critical source of data highlighting how COVID-19 racial/ethnic disparities have become ubiquitous [9].

Data released by the National Center for Health Statistics (NCHS) [10], initially in mid-May and recently updated to July 22, 2020, make it possible for the first time to explore with national data, using federally classified racial/ethnic groups, the likelihood that the Black, Hispanic, American Indian or Alaska Native, and Asian or Pacific Islander populations, in addition to experiencing higher COVID-19 mortality rates than the non-Hispanic White population, are also dying at younger ages.

## Methods

### Study population and data sources

The analytic plan, designed before data were reviewed or analyses began, was to quantify the age-specific COVID-19 mortality rates and to determine the magnitude of racial/ethnic disparities, in both relative and absolute terms. We did not create a formally documented analysis plan, but our a priori hypothesis was that these magnitudes would vary by age and that the relative magnitude would be greater at younger ages. In addition to computing rate ratios and rate differences, we also planned in advance to quantify the impact of COVID-19 mortality in relation to years of potential life lost (YPLL), using YPLL 65 and YPLL 75 (as a sensitivity analysis), and to quantify the racial/ethnic inequities in COVID-19 mortality rates using mortality risk ratios, risk differences, and premature mortality rates. The YPLL 75 analyses were included in the primary text at the request of reviewers.

These data were provided by the NCHS (https://data.cdc.gov). This analysis was based on data from https://data.cdc.gov/NCHS/Deaths-involvingcoronavirus-disease-2019-COVID-19/ks3g-spdg, accessed on July 22, 2020 [10], used rather than the data posted by the CDC COVID Data Tracker [11]. The NCHS data file includes death counts from New York City, a major initial hotspot for COVID-19, which is excluded in the CDC COVID Data Tracker, and also provides the data jointly (rather than separately) by race and ethnicity (Hispanic or not). Analysis of racial/ethnic groups was limited by the availability of denominator data in CDC WONDER [12] to non-Hispanic White (NHW), non-Hispanic Black (NHB), non-Hispanic American Indian or Alaska Native (NHAIAN), non-Hispanic Asian or Pacific Islander (NHAPI), and Hispanic. Only 0.9% of the NCHS COVID-19 deaths had missing data on race/ethnicity. Both the mortality data [10] and the denominator data [12] are publicly available de-identified datasets, and the data we used for our analyses are fully and freely available from the cited websites. Because this study used publicly available, nationally aggregated, de-identified mortality data, IRB review was not required.

Population data were the Vintage 2019 population estimates [12]. This study is reported as per the Strengthening the Reporting of Observational Studies in Epidemiology (STROBE) guideline (See S1 STROBE Checklist).

### Mortality rates, rate ratios, and rate differences

We calculated rates for 100,000 person-years by dividing deaths by the person-time from February 1 (the “Start Week” listed in the CDC data file) to July 22 (the “Data as of” field in the data file). This permits comparison of the age-specific and age-standardized rates to published mortality rates for common causes of death in previous years. We age-standardized to the Year 2000 standard million (used by the NCHS since 1999) and computed age-standardized rates, rate ratios, rate differences, and their confidence intervals using standard methods [13,14]. The methodology for computing directly age-standardized rates uses the following formula [14,15]:

$$R_{std}=\frac{\sum_{i}w_{i}r_{i}}{\sum_{i}w_{i}}$$

This approach weights the age-specific death rates (*r*_*i*_) by the US standard population age distribution (using 11 age categories: <1, 1–4, 5–14, …, 75–84, 85+ years), where *w*_*i*_ is the standard population for age group.

As an additional simple summary metric to quantify differential premature mortality risk [16,17], we calculated the age-standardized premature mortality rates, using age 65 and age 75 as cut points.

### YPLL and YPLL rates

To capture the population impact of premature death, we computed YPLL. This measure is calculated by multiplying the number of deaths in each age category by the number of years from the midpoint of the age category to a selected age cutoff, and summing over age (pp. 159–160 of [10]). The selection of any age cut point is arbitrary. Our goal was to capture years of life lost prematurely due to COVID-19, because we hypothesized that racial disparities for deaths at younger ages were especially marked. We used a cut point of 65 years because deaths before age 65 occur among people who are generally considered of working age and who are also more likely to leave dependent children than older adults. Additionally, 65 years corresponds to eligibility for Medicare and Social Security. To explore how robust our findings were using this cut point, we also examined a cut point of 75 years.

Because YPLL is sensitive to the size of the population and differences in the age distribution for racial/ethnic groups, we also computed the age-standardized YPLL rate per 100,000 persons by computing age-specific YPLL rates and then taking a weighted sum, with the weights coming from the Year 2000 standard million [16].

## Results

As of July 22, 2020, the number of COVID-19 deaths equaled 68,377 for NHW, 29,476 for NHB, 23,256 for Hispanic, 1,143 for NHAIAN, and 6,468 for NHAPI populations; the corresponding population sizes were 186.4 million, 40.6 million, 57.7 million, 2.6 million, and 19.5 million. (See S1 Table). For all racial/ethnic groups, the larger share of deaths occurred at older ages, although this proportion varies. For example, 10% of NHW deaths occurred before age 65 and 27% before age 75. In contrast, 28% and 54% of NHB deaths were before age 65 and age 75, respectively (See Table 1). Corresponding proportions for other groups are as follows: 37% and 61% for Hispanic, 45% and 69% for NHAIAN, and 23% and 49% for NHAPI.

**Table 1:** Crude, age-specific, and age-standardized COVID-19 mortality rates per 100,000 person-years for non-Hispanic White, non-Hispanic Black, Hispanic, non-Hispanic American Indian or Alaska Native, and non-Hispanic Asian or Pacific Islander populations, and age-specific mortality rate ratios and rate differences per 100,000 person-years.

Population	Deaths	Percent of deaths	Population	Percent of population	Age-specific mortality rate per 100,000 person-years (95% CI)	Rate ratio (95% CI)	*p*-Value	Rate difference per 100,000 person-years (95% CI)	*p*-Value
**Non-Hispanic White**									
Under 1 year	3	0.0%	1,994,440	1.1%	0.3 (0.1, 0.8)	1.0 (reference)		0.0 (reference)	
1–4 years	3	0.0%	8,244,087	4.4%	0.1 (0.0, 0.2)	1.0 (reference)		0.0 (reference)	
5–14 years	2	0.0%	21,483,759	11.5%	0.0 (0.0, 0.1)	1.0 (reference)		0.0 (reference)	
15–24 years	31	0.0%	23,544,616	12.6%	0.3 (0.2, 0.4)	1.0 (reference)		0.0 (reference)	
25–34 years	151	0.2%	25,657,465	13.8%	1.2 (1.1, 1.4)	1.0 (reference)		0.0 (reference)	
35–44 years	353	0.5%	23,709,326	12.7%	3.2 (2.8, 3.5)	1.0 (reference)		0.0 (reference)	
45–54 years	1,366	2.0%	26,232,985	14.1%	11.1 (10.5, 11.6)	1.0 (reference)		0.0 (reference)	
55–64 years	4,998	7.3%	15,189,511	8.1%	69.9 (67.9, 71.8)	1.0 (reference)		0.0 (reference)	
65–74 years	11,746	17.2%	23,091,706	12.4%	108.0 (106.1, 110.0)	1.0 (reference)		0.0 (reference)	
75–84 years	19,334	28.3%	12,034,203	6.5%	341.2 (336.4, 346.0)	1.0 (reference)		0.0 (reference)	
85 years and over	30,390	44.4%	5,223,448	2.8%	1,235.5 (1,221.6, 1,249.4)	1.0 (reference)		0.0 (reference)	
All ages—crude	68,377		186,405,546		36.7 (36.4, 37.0)	1.0 (reference)		0.0 (reference)	
All ages—age-standardized	68,377		186,405,546		49.9 (48.8, 51.0)	1.0 (reference)		0.0 (reference)	
Before 65 (age-standardized)	6,907	10.1%	146,056,189	78.4%	9.5 (8.9, 10.1)	1.0 (reference)		0.0 (reference)	
Before 75 (age standardized)	18,653	27.3%	169,147,895	90.7%	16.4 (15.8, 17.1)	1.0 (reference)		0.0 (reference)	
**Non-Hispanic Black**									
Under 1 year	2	0.0%	591,754	1.5%	0.7 (0.1, 2.0)	2.2 (0.4, 13.4)	0.188	0.4 (−0.7, 1.5)	0.230
1–4 years	1	0.0%	2,447,225	6.0%	0.1 (0.0, 0.3)	1.1 (0.1, 10.8)	0.460	0.0 (−0.2, 0.2)	0.461
5–14 years	8	0.0%	6,217,144	15.3%	0.3 (0.1, 0.5)	13.8 (2.9, 65.1)	<0.001	0.3 (0.1, 0.4)	0.005
15–24 years	58	0.2%	6,500,474	16.0%	1.9 (1.4, 2.4)	6.8 (4.4, 10.5)	<0.001	1.6 (1.1, 2.1)	<0.001
25–34 years	278	0.9%	6,658,091	16.4%	8.9 (7.8, 9.9)	7.1 (5.8, 8.7)	<0.001	7.6 (6.6, 8.7)	<0.001
35–44 years	723	2.5%	5,414,553	13.3%	28.4 (26.3, 30.4)	9.0 (7.9, 10.2)	<0.001	25.2 (23.1, 27.3)	<0.001
45–54 years	2,029	6.9%	5,287,236	13.0%	81.5 (77.9, 85.0)	7.4 (6.9, 7.9)	<0.001	70.4 (66.8, 74.0)	<0.001
55–64 years	4,997	17.0%	2,653,390	6.5%	399.9 (388.8, 411.0)	5.7 (5.5, 6.0)	<0.001	330.0 (318.8, 341.3)	<0.001
65–74 years	7,778	26.4%	3,006,666	7.4%	549.3 (537.1, 561.6)	5.1 (4.9, 5.2)	<0.001	441.3 (429.0, 453.7)	<0.001
75–84 years	7,705	26.1%	1,329,955	3.3%	1,230.3 (1,202.8, 1,257.7)	3.6 (3.5, 3.7)	<0.001	889.1 (861.2, 917.0)	<0.001
85 years and over	5,897	20.0%	507,505	1.2%	2,467.5 (2,404.5, 2,530.5)	2.0 (1.9, 2.0)	<0.001	1,232.0 (1,167.5, 1,296.5)	<0.001
All ages—crude	29,476		40,613,993		72.6 (71.7, 73.4)	2.0 (2.0, 2.0)	<0.001	35.9 (35.0, 36.8)	<0.001
All ages—age-standardized	29,476		40,613,993		181.7 (175.7, 187.8)	3.6 (3.5, 3.8)	<0.001	131.8 (125.7, 138.0)	<0.001
Before 65 (age-standardized)	8,096	27.5%	35,769,867	88.1%	59.5 (56.2, 62.9)	6.3 (5.8, 6.8)	<0.001	50.0 (46.6, 53.4)	<0.001
Before 75 (age standardized)	15,874	53.9%	38,776,533	95.5%	94.0 (89.9, 98.0)	9.9 (9.2, 10.6)	<0.001	84.4 (80.3, 88.5)	<0.001
**Hispanic or Latino**									
Under 1 year	5	0.0%	1,007,577	1.7%	1.1 (0.1, 2.0)	3.3 (0.8, 13.8)	0.051	0.7 (−0.3, 1.7)	0.073
1–4 years	4	0.0%	4,164,396	7.2%	0.2 (0.0, 0.4)	2.6 (0.6, 11.8)	0.102	0.1 (−0.1, 0.3)	0.128
5–14 years	5	0.0%	10,535,155	18.2%	0.1 (0.0, 0.2)	5.1 (1.0, 26.3)	0.026	0.1 (−0.0, 0.2)	0.043
15–24 years	83	0.4%	9,814,256	17.0%	1.8 (1.4, 2.2)	6.4 (4.2, 9.7)	<0.001	1.5 (1.1, 1.9)	<0.001
25–34 years	387	1.7%	9,429,166	16.3%	8.7 (7.8, 9.6)	7.0 (5.8, 8.4)	<0.001	7.5 (6.6, 8.4)	<0.001
35–44 years	1,122	4.8%	8,587,112	14.9%	27.7 (26.1, 29.4)	8.8 (7.8, 9.9)	<0.001	24.6 (22.9, 26.2)	<0.001
45–54 years	2,569	11.0%	7,025,565	12.2%	77.7 (74.6, 80.7)	7.0 (6.6, 7.5)	<0.001	66.6 (63.5, 69.7)	<0.001
55–64 years	4,487	19.3%	2,749,799	4.8%	346.5 (336.4, 356.6)	5.0 (4.8, 5.2)	<0.001	276.6 (266.3, 287.0)	<0.001
65–74 years	5,435	23.4%	2,682,684	4.6%	430.2 (418.8, 441.7)	4.0 (3.9, 4.1)	<0.001	322.2 (310.6, 333.8)	<0.001
75–84 years	5,106	22.0%	1,236,374	2.1%	877.0 (852.9, 901.0)	2.6 (2.5, 2.6)	<0.001	535.8 (511.3, 560.4)	<0.001
85 years and over	4,053	17.4%	499,028	0.9%	1,724.7 (1,671.6, 1,777.8)	1.4 (1.4, 1.4)	<0.001	489.2 (434.3, 544.1)	<0.001
All ages—crude	23,256		57,731,112		40.3 (39.8, 40.8)	1.1 (1.1, 1.1)	<0.001	3.6 (3.0, 4.2)	<0.001
All ages—age-standardized	23,256		57,731,112		141.2 (135.8, 146.5)	2.8 (2.7, 3.0)	<0.001	91.3 (85.8, 96.7)	<0.001
Before 65 (age-standardized)	8,662	37.2%	53,313,026	92.3%	53.4 (50.4, 56.4)	5.6 (5.2, 6.1)	<0.001	43.9 (40.9, 47.0)	<0.001
Before 75 (age standardized)	14,097	60.6%	55,995,710	97.0%	79.9 (76.2, 83.6)	8.4 (7.8, 9.1)	<0.001	70.4 (66.7, 74.1)	<0.001
Non-Hispanic American Indian or Alaska Native*									
Under 1 year	0	0.0%	38,260	1.5%	0.0 (0.0, 0.0)	—	—	−0.3 (−0.7, 0.0)	0.042
1–4 years	1	0.1%	156,473	6.0%	1.4 (0.0, 5.0)	17.6 (1.8, 168.8)	0.007	1.3 (−1.4, 3.9)	0.173
5–14 years	0	0.0%	409,393	15.8%	0.0 (0.0, 0.0)	—	—	−0.0 (−0.0, 0.0)	0.079
15–24 years	8	0.7%	419,255	16.2%	4.1 (1.2, 6.9)	14.5 (6.7, 31.5)	<0.001	3.8 (1.0, 6.6)	0.004
25–34 years	47	4.1%	418,797	16.2%	23.8 (17.0, 30.6)	19.1 (13.8, 26.5)	<0.001	22.6 (15.8, 29.4)	<0.001
35–44 years	70	6.1%	333,378	12.9%	44.6 (34.1, 55.0)	14.1 (10.9, 18.2)	<0.001	41.4 (31.0, 51.9)	<0.001
45–54 years	145	12.7%	326,384	12.6%	94.3 (79.0, 109.7)	8.5 (7.2, 10.1)	<0.001	83.3 (67.9, 98.7)	<0.001
55–64 years	245	21.4%	174,263	6.7%	298.6 (261.2, 335.9)	4.3 (3.8, 4.9)	<0.001	228.7 (191.2, 266.1)	<0.001
65–74 years	267	23.4%	202,493	7.8%	280.0 (246.4, 313.6)	2.6 (2.3, 2.9)	<0.001	172.0 (138.3, 205.6)	<0.001
75–84 years	219	19.2%	85,020	3.3%	547.0 (474.6, 619.4)	1.6 (1.4, 1.8)	<0.001	205.8 (133.2, 278.4)	<0.001
85 years and over	141	12.3%	28,950	1.1%	1,034.3 (863.5, 1,205.0)	0.8 (0.7, 1.0)	0.982	−201.2 (−372.5, −29.9)	0.011
All ages—crude	1,143		2,592,666		44.1 (41.5, 46.6)	1.2 (1.1, 1.3)	<0.001	7.4 (4.8, 10.0)	<0.001
All ages—age-standardized	1,143		2,592,666		108.9 (90.5, 127.4)	2.2 (1.8, 2.6)	<0.001	59.0 (40.5, 77.6)	<0.001
Before 65 (age-standardized)	516	45.1%	2,276,203	87.8%	57.1 (44.3, 69.9)	6.0 (4.7, 7.6)	<0.001	47.6 (34.7, 60.4)	<0.001
Before 75 (age standardized)	783	68.5%	2,478,696	95.6%	72.8 (58.6, 86.9)	7.6 (6.2, 9.4)	<0.001	63.2 (49.1, 77.4)	<0.001
**Non-Hispanic Asian or Pacific Islander**									
Under 1 year	1	0.0%	216,177	1.1%	1.0 (0.0, 3.6)	3.1 (0.3, 29.6)	0.165	0.7 (−1.3, 2.6)	0.254
1–4 years	0	0.0%	949,886	4.9%	0.0 (0.0, 0.0)	—	—	−0.1 (−0.2, 0.0)	0.958
5–14 years	1	0.0%	2,429,718	12.5%	0.1 (0.0, 0.3)	4.4 (0.4, 48.8)	0.112	0.1 (−0.1, 0.2)	0.222
15–24 years	7	0.1%	2,692,199	13.8%	0.6 (0.1, 1.0)	2.0 (0.9, 4.5)	0.052	0.3 (−0.1, 0.7)	0.102
25–34 years	58	0.9%	3,534,255	18.1%	3.5 (2.6, 4.4)	2.8 (2.1, 3.8)	<0.001	2.2 (1.3, 3.2)	<0.001
35–44 years	115	1.8%	3,233,519	16.6%	7.6 (6.2, 8.9)	2.4 (1.9, 3.0)	<0.001	4.4 (3.0, 5.8)	<0.001
45–54 years	373	5.8%	2,759,529	14.2%	28.7 (25.8, 31.6)	2.6 (2.3, 2.9)	<0.001	17.6 (14.7, 20.6)	<0.001
55–64 years	905	14.0%	1,174,022	6.0%	163.7 (153.0, 174.4)	2.3 (2.2, 2.5)	<0.001	93.8 (83.0, 104.7)	<0.001
65–74 years	1,518	23.5%	1,508,767	7.7%	213.7 (202.9, 224.4)	2.0 (1.9, 2.1)	<0.001	105.6 (94.7, 116.6)	<0.001
75–84 years	1,629	25.2%	708,822	3.6%	488.0 (464.3, 511.7)	1.4 (1.4, 1.5)	<0.001	146.9 (122.7, 171.0)	<0.001
85 years and over	1,861	28.8%	285,572	1.5%	1,383.9 (1,321.0, 1,446.7)	1.1 (1.1, 1.2)	<0.001	148.4 (84.0, 212.8)	<0.001
All ages—crude	6,468		19,492,466		33.2 (32.4, 34.0)	0.9 (0.9, 0.9)	<0.001	−3.5 (−4.4, −2.6)	<0.001
All ages—age-standardized	6,468		19,492,466		77.4 (71.9, 82.9)	1.6 (1.4, 1.7)	<0.001	27.5 (21.9, 33.1)	<0.001
Before 65 (age-standardized)	1,460	22.6%	16,989,305	87.2%	22.8 (19.8, 25.9)	2.4 (2.1, 2.8)	<0.001	13.3 (10.2, 16.5)	<0.001
Before 75 (age standardized)	2,978	46.0%	18,498,072	94.9%	36.3 (32.6, 39.9)	3.8 (3.4, 4.3)	<0.001	26.7 (23.0, 30.4)	<0.001

*Caution is required regarding the Non-Hispanic American Indian/Alaska Native data, which have a well-known problem with accuracy. See [19–21].

**Fig 1:**
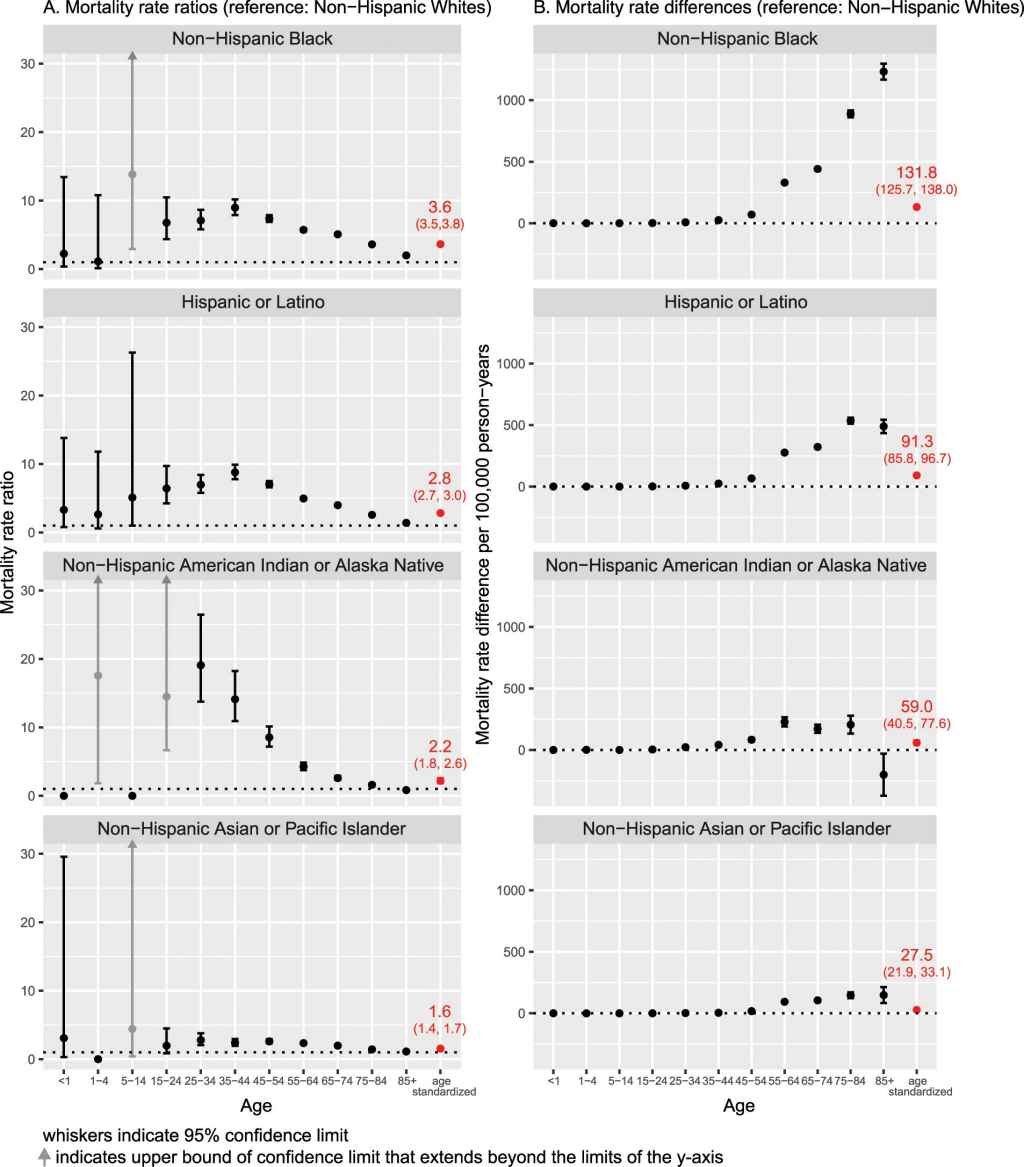
Age-specific and age-standardized COVID-19 mortality rate ratios and rate differences per 100,000 person-years with 95% confidence intervals (reference: non-Hispanic White population), as of July 22, 2020, United States. (A) Mortality rate ratios. (B) Mortality rate differences.

Fig 1 shows the racial/ethnic disparities in COVID-19 mortality by age categories as reflected in rate ratios and risk differences, with the NHW population as the reference, along with the age-standardized rate. For data on all-cause mortality, see S1 Table.

### Relative rate ratios

Discounting patterns for ages below 25 years, where the small numbers of deaths mean rate estimates are unstable, Fig 1A shows that there are racial/ethnic disparities, relative to the NHW population, in every age stratum. Among the NHB, Hispanic, and NHAIAN populations, these relative gaps widen and become especially stark among young adults into midlife (persons aged 25–54 years). NHB rate ratios relative to NHW were as high as 7.1 (95% CI 5.8, 8.7; *p <* 0.001) for persons aged 25–34 years, 9.0 (95% CI 7.9, 10.2; *p <* 0.001) for persons aged 35–44 years, and 7.4 (95% CI 6.9, 7.9; *p <* 0.001) for persons aged 45–54 years. Even at older ages, NHB rate ratios were between 2.0 and 5.7. Similarly, rate ratios for the Hispanic versus NHW population were 7.0 (95% CI 5.8, 8.4; *p <* 0.001), 8.8 (95% CI 7.8, 9.9; *p <* 0.001), and 7.0 (95% CI 6.6, 7.5; *p <* 0.001) for the corresponding age strata above, with remaining rate ratios ranging from 1.4 to 5.0. Rate ratios for the NHAIAN population were similarly high through age 84 years. Among NHAPI persons, rate ratios ranged from 2.0 to 2.8 for persons aged 25–74 years and were 1.4 and 1.1 for persons aged 75–84 and 85+ years, respectively. By contrast, the age-standardized rate ratios equaled 3.6 (95% CI 3.5, 3.8; *p <* 0.001) for NHB, 2.8 (95% CI 2.7, 3.0; *p <* 0.001) for Hispanic, 2.2 (1.8, 2.6; *p <* 0.001) for NHAIAN, and 1.6 (1.4, 1.7; *p <* 0.001) for NHAPI populations (see Table 1).

### Rate differences

Fig 1B displays the racial/ethnic mortality rate differences, an absolute measure, for each age stratum, as compared to the NHW population. For all groups, these differences compared to the NHW population increased with advancing age and were especially pronounced for the NHB population. For example, among persons aged 75–84 years, the mortality rate difference equaled 889.1 (95% CI 861.2, 917.0; *p <* 0.001) per 100,000 person-years for NHB, 535.8 (95% CI 511.3, 560.4; *p <* 0.001) for Hispanic, 205.8 (95% CI 133.2, 278.4; *p <* 0.001) for NHAIAN, and 146.9 (95% CI 122.7, 171.0; *p <* 0.001) for NHAPI populations.

Finally, we examined whether the age-standardized premature COVID-19 mortality rates per 100,000 person-years reflect the racial/ethnic disparity in risk of early death, either before age 65 or before age 75. The age-standardized premature mortality (before age 65) per 100,000 person-years was 9.5 (95% CI 8.9, 10.1) for NHW, 59.5 (95% CI 56.2, 62.9) for NHB, 53.4 (95% CI 50.4, 56.4) for Hispanic, 57.1 (95% CI 44.3, 69.9) for NHAIAN, and 22.8 (95% CI 19.8, 25.9) for NHAPI populations. Similarly, for premature death before age 75, the age-standardized rates per 100,000 person-years were as follows: NHW, 16.4 (95% CI 15.8, 17.1); NHB, 94.0 (95% CI 89.9, 98.0); Hispanic, 79.9 (95% CI 76.2, 83.6); NHAIAN, 72.8 (95% CI 58.6, 86.9); and NHAPI, 36.3 (95% CI 32.6, 39.9) (see Table 1).

Table 2 shows the corresponding YPLL for COVID-19 (see S2 Table showing YPLL for all-cause mortality, for comparison). The disparities in COVID-19 mortality translate to 86,466 (95% CI 84,043 to 88,889) YPLL before age 65 (YPLL 65) for NHB, 107,146 (95% CI 104,315 to 109,976) for Hispanic, 7,217 (95% CI 6,433 to 8,001) for NHAIAN, and 15,460 (95% CI 14,439 to 16,480) for NHAPI populations, compared with 61,474 (95% CI 59,584 to 63,365) for the NHW population. Accounting for age distribution and population size differences between racial/ethnic groups, the age-standardized YPLL rate ratio, compared to the NHW population, equaled 6.9 (95% CI 6.9, 6.9; *p <* 0.001) for NHB, 6.9 (95% CI 6.8, 6.9; *p <* 0.001) for Hispanic, 8.7 (95% CI 8.6, 8.7; *p <* 0.001) for NHAIAN, and 2.5 (95% CI 2.4, 2.47; *p <* 0.001) for NHAPI.

**Table 2:** Years of potential life lost (YPLL) and age-standardized YPLL rates per 100,000 persons due to COVID-19 using age 65 and age 75 as cutoffs, with age-standardized YPLL ratios compared to the non-Hispanic White population as of July 22, 2020, United States.

Measure	Non-Hispanic White	Non-Hispanic Black	Hispanic or Latino	Non-Hispanic American Indian or Alaska Native*	Non-Hispanic Asian or Pacific Islander
YPLL65	61,474 (59,584, 63,365)	86,466 (84,043, 88,889)	107,146 (104,315, 109,976)	7,217 (6,433, 8,001)	15,460 (14,439, 16,480)
YPLL65 difference (compared to Non-Hispanic White)	0 (reference)	24,992 (21,918, 28,065)	−54,258 (−56,305, −52,210)	−46,015 (−48,163, −43,867)	45,671 (42,267, 49,075)
Age-standardized YPLL65 rate per 100,000 persons	34.9 (31.7, 38.0)	240.9 (222.6, 259.2)	224.2 (209.1, 239.3)	303.9 (213.4, 394.4)	85.6 (70.9, 100.2)
Age-standardized YPLL65 rate ratio (compared to non-Hispanic White)	1.00 (reference)	6.9 (6.9, 6.9)	6.4 (6.4, 6.4)	8.7 (8.6, 8.9)	2.5 (2.4, 2.5)
YPLL75	189,274 (185,799, 192,750)	206,316 (202,270, 210,362)	220,940 (216,458, 225,423)	13,712 (12,532, 14,892)	37,650 (35,936, 39,363)
YPLL75 difference (compared to non-Hispanic-White)	0 (reference)	17,042 (11,708, 22,375)	−175,562 (−179,233, −171,892)	−151,625 (−155,500, −147,750)	31,666 (25,994, 37,338)
Age-standardized YPLL75 rate per 100,000 persons	90.9 (85.3, 96.4)	571.2 (539.0, 603.5)	510.9 (483.2, 538.6)	582.4 (440.1, 724.6)	212.8 (185.3, 240.3)
Age-standardized YPLL75 rate ratio (compared to non-Hispanic White)	1.00 (reference)	6.3 (6.3, 6.3)	5.6 (5.6, 5.6)	6.4 (6.3, 6.5)	2.3 (2.3, 2.4)

95% CIs given in parentheses.

*Caution is required regarding the Non-Hispanic American Indian/Alaska Native data, which have a well-known problem with accuracy. See [19–21].

Because there were relatively few deaths below age 65, ranging from 10% of all deaths for the NHW population to 45% of deaths among NHAIAN persons, a numerically small group with a population of about 2.6 million, we also examined YPLL75, because the number of deaths before age 75 is much larger. YPLL75 for all racial/ethnic groups was 2- to 3-fold larger than YPLL65, as expected because risk of COVID-19 death—and all death—increases with age. The YPLL75 rate ratios by race/ethnicity were similar to those for YPLL65, albeit slightly attenuated, and equaled, for NHB (6.3; 95% CI 6.3 to 6.33; *p <* 0.001), Hispanic (5.62; 95% CI 5.6 to 5.7; *p <* 0.001), NHAIAN (6.4; 95% CI 6.30 to 6.5; *p <* 0.001), and NHAPI (2.3; 95% CI 2.3 to 2.3; *p <* 0.001).

## Discussion

We used newly available public data on COVID-19 deaths to analyze patterns of age-specific mortality by race/ethnicity. The main study finding is an excess risk of COVID-19 death at all ages in the NHB, NHAIAN, and NHAPI populations as compared to the NHW population. Disparities were particularly extreme at younger ages (25–54 years old). We additionally went beyond the computation and comparison of age-specific mortality rates to assess the differential burden of COVID-19 mortality in relation to both premature mortality and YPLL. The impact of lives prematurely cut short (before attaining 65 years) can be measured in the absolute number of YPLL. For both the NHB and Hispanic populations, this loss is much larger than for the NHW population—despite the fact that the NHW population is larger—with a ratio of is 4.6:1 and 3.2:1, respectively, for the NHB and Hispanic populations. Poor quality of NHAIAN mortality and population data likely means the estimated excesses are underestimates [18–21]. Although for all groups by far the majority of deaths occur above the age of 65 years, premature deaths deprive people of their anticipated life expectancy. As a consequence, the NHB and Hispanic populations lost nearly 7 times and the NHAIAN population nearly 9 times as many years of life before the age of 65 as did the NHW population.

Examination of age-specific mortality rates, and not simply counts of deaths or crude comparisons of the racial/ethnic composition of COVID-19 deaths to the total population, is crucial to revealing racial/ethnic disparities. Age-standardized rates are not sufficient because age standardization, while accounting for the different age distributions across racial/ethnic groups, notably obscure the magnitude of mortality inequities at younger ages [6–8]. These COVID-19 mortality rate ratios, 7- to 9-fold higher for the NHB, NHAIAN, and Hispanic populations, are extreme and reflect the devastating toll COVID-19 has taken among communities of color. To put these extreme rates in context, in 2017, the rate ratios for all-cause mortality comparing the US NHB to NHW populations, by 5-year groups for persons aged 25–29 years up through aged 60–64 years, ranged between 1.3 and 1.5 [22]. Additionally, in 2015, the age-standardized rate ratio for premature death (before age 65) among adults aged 20–64 years, comparing the US NHB to NHW population, equaled 1.5 for all-cause mortality, and ranged between 1.1 to at most 2.2 for specific causes of mortality, including leading causes of death (circulatory, neoplasms, endocrine [including diabetes], and respiratory) [7].

To capture the magnitude of racial/ethnic inequities of COVID-19, age-specific mortality rates for COVID-19 should be routinely available by race/ethnicity as well as by sex. Grasping the disparate impact of this pandemic requires transparent reporting of not only age-specific rate ratios and rate differences, but also YPLL. Robust evidence documents the transgenerational adverse impacts of parental death at younger ages on their children’s economic and health trajectories [23–25]. Our data underscore that COVID-19 will likely exacerbate these harms.

This study has several limitations. NCHS data are based on the completed death certificates received by the CDC, and thus may lag in capturing the total number of deaths compared to what is reported on state dashboards [9]. However, this lag likely would lead to underestimates of YPLL (due to deaths not yet included). Further, reporting of deaths due to COVID-19 depends in part on the accessibility of COVID-19 tests, a problem that would likely introduce a conservative bias for our racial/ethnic comparisons, given evidence of reduced access to testing among US populations of color [26,27]. Another limitation is that analysis solely of deaths classified as due to COVID-19 do not capture the full excess burden of mortality due to the pandemic. At issue is not only potential misclassification of deaths but also deaths not directly due to SARS-COV-2 infection but nevertheless due to the pandemic, e.g., deaths due to people not seeking care for chest pains because of fear of viral exposure at hospitals [28–30]. Well-known problems affecting counts of NHAIAN deaths and the NHAIAN population likely lead to conservative estimates of risk [18–21], but misclassification of race/ethnicity on death certificates is very low for the remaining racial/ethnic groups [18].

The mortality data we report do not reveal why excess deaths occur among US populations of color compared to the US NHW population, but any explanation must account for these observed age-specific patterns, also replicated in recently released data from Massachusetts [31]. Mortality rates reflect both the incidence of COVID-19 and its case fatality rate. Higher rates of co-morbid conditions among young adults in the NHB and Hispanic populations would contribute to enhanced mortality risk, as would more limited adoption of social distancing in communities of color and lack of access to healthcare. However, the extremely high age-specific mortality rate ratios we report for working-age adults, ranging between 5 and 9, cannot plausibly be explained as due mainly to co-morbid conditions. While many common conditions show racial/ethnic disparities, the relative excess in the NHB and Hispanic populations is far lower than reported here for COVID-19 mortality, with risk ratios typically under or close to 2. For example, in the case of NHB versus NHW comparisons among adults under age 65, the rate ratios for mortality due to cardiovascular disease, cancer, and diabetes are all under 2 [7], as are the rate ratios for prevalence of obesity [32,33].

In addition, continued young adult participation in the workforce outside the home increases risk of exposure to SARS-CoV-2. Data suggest that communities with a high proportion of service workers [34] and communities with cell phone mobility patterns consistent with work-related commutes and overall mobility [35,36] also have higher prevalence of COVID-19. A community prevalence study in the Mission District of San Francisco showed that 90% of those with positive viral tests were working outside the home [37]. Together, these observations, while not conclusive, support concern that low-wage essential workers are not adequately protected in the workplace. While higher rates of chronic disease and barriers to healthcare access can be addressed by policy interventions, approaches that lower viral exposure would have an immediate short-term impact. Possible actions include permitting workers to stay home when personal or household members’ health creates a higher risk for poor outcome, offering hazard pay, paid sick leave, health insurance, personal protective equipment, access to handwashing, and social distancing at work, along with, for those who so choose, alternative accommodation.

## Supporting information

S1 STROBE Checklist Checklist for cross-sectional studies. (DOCX)

S1 Table Crude, age-specific, and age-standardized all-cause mortality rates per 100,000 person-years for non-Hispanic White, non-Hispanic Black, Hispanic, non-Hispanic American Indian or Alaska Native, and non-Hispanic Asian or Pacific Islander populations, and age-specific mortality rate ratios and rate differences per 100,000 person-years. (DOCX)

S2 Table Years of potential life lost (YPLL) and age-standardized YPLL rates per 100,000 persons due to all-cause mortality using age 65 and age 75 as cutoffs, with age-standardized YPLL ratios compared to the non-Hispanic White population as of July 22, 2020, United States. (DOCX)

## Abbreviations

CDC: Centers for Disease Control and Prevention
NCHS: National Center for Health Statistics
NHAIAN: non-Hispanic American Indian/Alaska Native
NHAPI: non-Hispanic Asian or Pacific Islander
NHB: non-Hispanic Black
NHW: non-Hispanic White
YPLL: years of potential life lost
