# Correction: Variation in racial/ethnic disparities in COVID-19 mortality by age in the United States: A cross-sectional study

**DOI:** 10.1371/journal.pmed.1003541

**Published:** 2021-02-04

**Authors:** Mary T. Bassett, Jarvis T. Chen, Nancy Krieger

There are several transcription errors in the text.

The third sentence of the subsection of the Abstract titled “Methods and Findings” should read: “As of July 22, 2020, the number of COVID-19 deaths equaled 68,377 for NHW, 29,476 for NHB, 23,256 for Hispanic, 1,143 for NHAIAN, and 6,468 for NHAPI populations; the corresponding population sizes were 186.4 million, 40.6 million, 57.7 million, 2.6 million, and 19.5 million.”

The sixth sentence of the subsection of the Abstract titled “Methods and Findings” should read: “Similarly, rate ratios for the Hispanic versus NHW population were 7.0 (95%CI 5.8, 8.4; p<0.001), 8.8 (95% CI 7.8, 9.9; p < 0.001), and 7.0 (95% CI 6.6, 7.5; p < 0.001) for the corresponding age strata above, with remaining rate ratios ranging from 1.4 to 5.0.” The eighth sentence in the same section should read: “Among NHAPI persons, rate ratios ranged from 2.0 to 2.8 for persons 25–74 years and were 1.4 and 1.1 for persons aged 75–84 and 85+ years, respectively.”

The final sentence of the first paragraph of the Results should read: “Corresponding proportions for other groups are as follows: 37% and 61% for Hispanic, 45% and 69% for NHAIAN, and 23% and 46% for NHAPI.”

The third sentence of the third paragraph within the subsection of the Results titled “Rate differences” should read: “Accounting for age distribution and population size differences between racial/ethnic groups, the age-standardized YPLL rate ratio, compared to the NHW population, equaled 6.9 (95% CI 6.9, 6.9; *p <*0.001) for NHB, 6.4 (95% CI 6.4, 6.4; *p <* 0.001) for Hispanic, 8.7 (95% CI 8.6, 8.9; *p <* 0.001) for NHAIAN, and 2.5 (95% CI 2.4, 2.5; *p <* 0.001) for NHAPI.”

There are several transcriptions errors in the Table 2.

In Line 2 of Table 2, “YPLL65 (compared to non-Hispanic White)”, under the column labeled “Hispanic or Latino” the numbers should read 45,671 (42,267, 49,075); under “Non-Hispanic American Indian or Alaska Native”, the numbers should read -54,258 (-56,305, -52,210); under “Non-Hispanic Asian or Pacific Islander”, the numbers should read -46,015 (-48,163, -43,867).

In Line 6 of Table 2 “YPLL75 difference (compared to non-Hispanic White)”, under the column labeled “Hispanic or Latino” the numbers should read 31,666 (25,994, 37,338); under “Non-Hispanic American Indian or Alaska Native”, the numbers should read -175,562 (-179,233, -171,892); under “Non-Hispanic Asian or Pacific Islander”, the numbers should read -151,625 (-155,500, -147,750).

Please see corrected Table 2.

**Table 2:** Years of Potential Life Lost (YPLL) and age-standardized YPLL rates per 100,000 due to COVID-19 using age 65 and age 75 as cutoffs, with age-standardized YPLL ratios compared to Non-Hispanic whites as of July 22, 2020, United States.

	Non-Hispanic White	Non-Hispanic Black	Hispanic or Latino	Non-Hispanic American Indian or Alaska Native	Non-Hispanic Asian or Pacific Islander
**COVID-19**					
YPLL65	61474 (59584, 63365)	86466 (84043, 88889)	107146 (104315, 109976)	7217 (6433, 8001)	15460 (14439, 16480)
YPLL65 difference (compared to Non-Hispanic White)	0 (reference)	24992 (21918, 28065)	45671 (42267, 49075)	-54258 (-56305, -52210)	-46015 (-48163, -43867)
Age-standardized YPLL65 rate per 100,000	34.9 (31.7, 38.0)	240.9 (222.6, 259.2)	224.2 (209.1, 239.3)	303.9 (213.4, 394.4)	85.6 (70.9, 100.2)
Age-standardized YPLL65 rate ratio (compared to Non-Hispanic White)	1.00 (reference)	6.9 (6.9, 6.9)	6.4 (6.4, 6.4)	8.7 (8.6, 8.9)	2.5 (2.4, 2.5)
YPLL75	189274 (185799, 192750)	206316 (202270, 210362)	220940 (216458, 225423)	13712 (12532, 14892)	37650 (35936, 39363)
YPLL75 difference (compared to Non-Hispanic-White)	0 (reference)	17042 (11708, 22375)	31666 (25994, 37338)	-175562 (-179233, -171892)	-151625 (-155500, -147750)
Age-standardized YPLL75 rate per 100,000	90.9 (85.3, 96.4)	571.2 (539.0, 603.5)	510.9 (483.2, 538.6)	582.4 (440.1, 724.6)	212.8 (185.3, 240.3)
Age-standardized YPLL75 rate ratio (compared to Non-Hispanic White)	1.00 (reference)	6.3 (6.3, 6.3)	5.6 (5.6, 5.6)	6.4 (6.3, 6.5)	2.3 (2.3, 2.4)

There are several transcription errors in S2 Table.

In Line 2 of S2 Table, “YPLL65 difference (compared to non-Hispanic White) under the column labeled “Hispanic or Latino” the numbers should read -2261828 (-2282862, -2240795); under “Non-Hispanic American Indian or Alaska Native”, the numbers should read -3123128 (-3141399, -3104858); under “Non-Hispanic Asian or Pacific Islander”, the numbers should read -3043196 (-3061704, -3024687).

In Line 6 of S2 Table, “YPLL75 difference (compared to non-Hispanic-White)” under the column labeled “Hispanic or Latino” the numbers should read -4963574 (-4993415, -4933732); under “Non-Hispanic American Indian or Alaska Native”, the numbers should read -6386508 (-6412895, -6360122); under “Non-Hispanic Asian or Pacific Islander”, the numbers should read -6217010 (-6243731, -6190290).

Please see corrected S2 Table.

## Supporting information

S2 Table Years of potential life lost (YPLL) and age-standardized YPLL rates per 100,000 persons due to all-cause mortality using age 65 and age 75 as cutoffs, with age-standardized YPLL ratios compared to the non-Hispanic White population as of July 22, 2020, United States. (DOCX)
