# Supplementary material for: Variation in racial/ethnic disparities in COVID-19 mortality by age in the United States: A cross-sectional study
Source: PLoS Med. 2020 Oct 20;17(10):e1003402. doi: 10.1371/journal.pmed.1003402 (PMC7575091; doi:10.1371/journal.pmed.1003402)
Supplement: S1 STROBE Checklist — (DOCX) [file pmed.1003402.s001.docx]

STROBE Statement—Checklist of items that should be included in reports of ***cross-sectional studies***

|  | Item No | Recommendation | Page No |
| --- | --- | --- | --- |
| **Title and abstract** | 1 | (*a*) Indicate the study’s design with a commonly used term in the title or the abstract | Title page: subtitle states “A cross-sectional study” |
|  |  | (*b*) Provide in the abstract an informative and balanced summary of what was done and what was found | Abstract provides a balanced informative summary |
| Introduction | | | |
| Background/rationale | 2 | Explain the scientific background and rationale for the investigation being reported | Provided in both the abstract and the introduction to the paper (text ¶1-2) |
| Objectives | 3 | State specific objectives, including any prespecified hypotheses | As reported in text ¶3, specific objectives and pre-specified hypotheses are provided in the Methods section. |
| Methods | | | |
| Study design | 4 | Present key elements of study design early in the paper | As reported in text ¶3-9, the methods section for our cross-sectional study presents: the data sources, time period and results. |
| Setting | 5 | Describe the setting, locations, and relevant dates, including periods of recruitment, exposure, follow-up, and data collection | As reported in text ¶3-4, the study setting for our cross-sectional study is the United States, and we employ national data on all COVID-19 deaths reported by the US Centers for Disease Control and Prevention from February 1, 2020 – July 22, 2020 |
| Participants | 6 | (*a*) Give the eligibility criteria, and the sources and methods of selection of participants | As reported in text ¶4, this study uses publicly available national data from the National Center for Health Statistics (for COVID-19 deaths) and US Census (for denominator data). Cases comprised deaths due attributed COVID-19 with race/ethnicity as reported to state and NYC health departments. |
| Variables | 7 | Clearly define all outcomes, exposures, predictors, potential confounders, and effect modifiers. Give diagnostic criteria, if applicable | As reported in text ¶4, variables are confined to age group and racial/ethnic group as described in the 2^nd^ para of the Methods |
| Data sources/ measurement | 8* | For each variable of interest, give sources of data and details of methods of assessment (measurement). Describe comparability of assessment methods if there is more than one group | As reported in text ¶4, this analysis was based on publicly available data collected by the Centers for Disease Control and Prevention and the US Census, and references to further details on these data are provided with the text. |
| Bias | 9 | Describe any efforts to address potential sources of bias | As reported in text ¶20, potential sources of bias due to under diagnosis and under reporting of COVID -19 are discussed in the Discussion section |
| Study size | 10 | Explain how the study size was arrived at | As reported in text ¶4, study size was determined by the total number of reported COVID-19 deaths during the study interval and the size of the population in the US from which these cases arose. |
| Quantitative variables | 11 | Explain how quantitative variables were handled in the analyses. If applicable, describe which groupings were chosen and why | As reported in text ¶6-9, age was grouped into a standard 11 categories, mainly in 10-year groups. The text explains that our interest was in premature mortality, assessed as <65, or <75 in the Methods section. We employed the racial/ethnic categories reported by the CDC, which are in compliance with the OMB regulations for reporting race/ethnicity in federal data. |
| Statistical methods | 12 | (*a*) Describe all statistical methods, including those used to control for confounding | As reported in text ¶6-9, these standard methods are described in the Methods section, with references provided |
|  |  | (*b*) Describe any methods used to examine subgroups and interactions | As reported in text ¶2, this analysis examined racial/ethnic groups as defined in the US Census, in compliance with the OMB regulations for reporting race/ethnicity in federal data. |
|  |  | (*c*) Explain how missing data were addressed | As reported in text ¶4, the proportion of missing data on racial/ethnic classification is given in the methods section. A very small proportion of data are missing this information (0.9%). |
|  |  | (*d*) If applicable, describe analytical methods taking account of sampling strategy | Not relevant |
|  |  | (*e*) Describe any sensitivity analyses | As reported in text ¶4 and 16, in the methods section and results we explain that looked racial variation used using at age cut points at 65 years as our primary analytic approach, and 75 years for our sensitivity analyses. |
| Results | | | |
| Participants | 13* | (a) Report numbers of individuals at each stage of study—eg numbers potentially eligible, examined for eligibility, confirmed eligible, included in the study, completing follow-up, and analysed | As reported in text ¶10, we provide these data in the results section |
|  |  | (b) Give reasons for non-participation at each stage | Not relevant |
|  |  | (c) Consider use of a flow diagram | Not relevant |
| Descriptive data | 14* | (a) Give characteristics of study participants (eg demographic, clinical, social) and information on exposures and potential confounders | As reported in text ¶10, we provide these data in the results section |
|  |  | (b) Indicate number of participants with missing data for each variable of interest | As reported in text ¶10, we provide these data in the results section |
| Outcome data | 15* | Report numbers of outcome events or summary measures | As reported in text ¶10, we provide these data in the results section |
| Main results | 16 | (*a*) Give unadjusted estimates and, if applicable, confounder-adjusted estimates and their precision (eg, 95% confidence interval). Make clear which confounders were adjusted for and why they were included | As reported in S1 Table, we provide the age standardized and crude estimates of mortality rates. |
|  |  | (*b*) Report category boundaries when continuous variables were categorized | Not relevant |
|  |  | (*c*) If relevant, consider translating estimates of relative risk into absolute risk for a meaningful time period | As reported in text ¶12-14, we provide both relative ratios and absolute rate differences for data collected February-July 2020 |
| Other analyses | 17 | Report other analyses done—eg analyses of subgroups and interactions, and sensitivity analyses | As reported in text ¶9 and 16, in the results section we provide the findings of our sensitivity analyses, which used age 75 as the cut-point for defining premature mortality (as compared to age 65). |
| Discussion | | | |
| Key results | 18 | Summarise key results with reference to study objectives | As provided in the abstract, key summary, and text ¶17, key results are summarized in relation to the study objectives. |
| Limitations | 19 | Discuss limitations of the study, taking into account sources of potential bias or imprecision. Discuss both direction and magnitude of any potential bias | As provided in text ¶20, we discuss study limitations in the discussion section. |
| Interpretation | 20 | Give a cautious overall interpretation of results considering objectives, limitations, multiplicity of analyses, results from similar studies, and other relevant evidence | As provided in text ¶20-22, we provide a cautious overall interpretation of study results and consider relevant limitations, results from other studies, and other relevant considerations. |
| Generalisability | 21 | Discuss the generalisability (external validity) of the study results | Our study is based on the available national data for the US, so generalizability is not relevant |
| Other information | | | |
| Funding | 22 | Give the source of funding and the role of the funders for the present study and, if applicable, for the original study on which the present article is based | Provided in the declaration to journal. |

*Give information separately for exposed and unexposed groups.

**Note:** An Explanation and Elaboration article discusses each checklist item and gives methodological background and published examples of transparent reporting. The STROBE checklist is best used in conjunction with this article (freely available on the Web sites of PLoS Medicine at http://www.plosmedicine.org/, Annals of Internal Medicine at http://www.annals.org/, and Epidemiology at http://www.epidem.com/). Information on the STROBE Initiative is available at www.strobe-statement.org.
