# Supplementary material for: Variation in racial/ethnic disparities in COVID-19 mortality by age in the United States: A cross-sectional study
Source: PLoS Med. 2020 Oct 20;17(10):e1003402. doi: 10.1371/journal.pmed.1003402 (PMC7575091; doi:10.1371/journal.pmed.1003402)
Supplement: S1 Table — (DOCX) [file pmed.1003402.s002.docx]

S1 Table: Crude, age-specific, and age-standardized all-cause mortality rates per 100,000 person-years for Non-Hispanic White, Non-Hispanic Black, Hispanic, Non-Hispanic American Indian or Alaskan Native, and Non-Hispanic Asian or Pacific Islander populations, and age-specific mortality rate ratios and rate differences per 100,000 person-years

| Population | Deaths | % of deaths | Population | % of population | Age-specific mortality rate per 100,000 person-years (95% CI) | Rate ratio (95% CI) | p-value | Rate difference per 100,000 person-years (95% CI) | p-value |
| --- | --- | --- | --- | --- | --- | --- | --- | --- | --- |
|  |  |  |  |  |  |  |  |  |  |
| **Non-Hispanic White** |  |  |  |  |  |  |  |  |  |
| Under 1 year | 3,301 | 0.3% | 1,994,440 | 1.1% | 351.5 (339.5, 363.5) | 1.0 (reference) |  | 0.0 (reference) |  |
| 1-4 years | 680 | 0.1% | 8,244,087 | 4.4% | 17.5 (16.2, 18.8) | 1.0 (reference) |  | 0.0 (reference) |  |
| 5-14 years | 1,140 | 0.1% | 21,483,759 | 11.5% | 11.3 (10.6, 11.9) | 1.0 (reference) |  | 0.0 (reference) |  |
| 15-24 years | 6,964 | 0.6% | 23,544,616 | 12.6% | 62.8 (61.3, 64.3) | 1.0 (reference) |  | 0.0 (reference) |  |
| 25-34 years | 16,982 | 1.6% | 25,657,465 | 13.8% | 140.6 (138.4, 142.7) | 1.0 (reference) |  | 0.0 (reference) |  |
| 35-44 years | 24,909 | 2.3% | 23,709,326 | 12.7% | 223.1 (220.3, 225.9) | 1.0 (reference) |  | 0.0 (reference) |  |
| 45-54 years | 48,794 | 4.5% | 26,232,985 | 14.1% | 395.0 (391.5, 398.5) | 1.0 (reference) |  | 0.0 (reference) |  |
| 55-64 years | 125,463 | 11.7% | 15,189,511 | 8.1% | 1754.0 (1744.3, 1763.7) | 1.0 (reference) |  | 0.0 (reference) |  |
| 65-74 years | 207,260 | 19.3% | 23,091,706 | 12.4% | 1906.0 (1897.8, 1914.2) | 1.0 (reference) |  | 0.0 (reference) |  |
| 75-84 years | 274,190 | 25.5% | 12,034,203 | 6.5% | 4838.3 (4820.2, 4856.4) | 1.0 (reference) |  | 0.0 (reference) |  |
| 85 years and over | 366,526 | 34.1% | 5,223,448 | 2.8% | 14900.8 (14852.5, 14949.0) | 1.0 (reference) |  | 0.0 (reference) |  |
| All ages - crude | 1,076,209 |  | 186,405,546 |  | 577.3 (576.3, 578.4) | 1.0 (reference) |  | 0.0 (reference) |  |
| All ages - age-standardized | 1,076,209 |  | 186,405,546 |  | 851.7 (846.9, 856.5) | 1.0 (reference) |  | 0.0 (reference) |  |
| Before 65 (age-standardized) | 228,233 | 21.2% | 146,056,189 | 78.4% | 318.0 (314.6, 321.4) | 1.0 (reference) |  | 0.0 (reference) |  |
| Before 75 (age standardized) | 435,493 | 40.5% | 169,147,895 | 90.7% | 429.6 (425.9, 433.3) | 1.0 (reference) |  | 0.0 (reference) |  |
|  |  |  |  |  |  |  |  |  |  |
| **Non-Hispanic Black** |  |  |  |  |  |  |  |  |  |
| Under 1 year | 2,241 | 1.1% | 591,754 | 1.5% | 804.2 (770.9, 837.5) | 2.3 (2.2, 2.4) | p<0.001 | 452.7 (417.3, 488.1) | p<0.001 |
| 1-4 years | 374 | 0.2% | 2,447,225 | 6.0% | 32.5 (29.2, 35.7) | 1.9 (1.6, 2.1) | p<0.001 | 14.9 (11.4, 18.5) | p<0.001 |
| 5-14 years | 542 | 0.3% | 6,217,144 | 15.3% | 18.5 (17.0, 20.1) | 1.6 (1.5, 1.8) | p<0.001 | 7.2 (5.6, 8.9) | p<0.001 |
| 15-24 years | 3,737 | 1.9% | 6,500,474 | 16.0% | 122.1 (118.2, 126.0) | 1.9 (1.9, 2.0) | p<0.001 | 59.3 (55.1, 63.5) | p<0.001 |
| 25-34 years | 6,879 | 3.5% | 6,658,091 | 16.4% | 219.4 (214.2, 224.6) | 1.6 (1.5, 1.6) | p<0.001 | 78.8 (73.2, 84.4) | p<0.001 |
| 35-44 years | 8,996 | 4.6% | 5,414,553 | 13.3% | 352.8 (345.5, 360.1) | 1.6 (1.5, 1.6) | p<0.001 | 129.7 (121.9, 137.5) | p<0.001 |
| 45-54 years | 16,681 | 8.5% | 5,287,236 | 13.0% | 670.0 (659.8, 680.1) | 1.7 (1.7, 1.7) | p<0.001 | 275.0 (264.2, 285.7) | p<0.001 |
| 55-64 years | 35,694 | 18.1% | 2,653,390 | 6.5% | 2856.6 (2827.0, 2886.3) | 1.6 (1.6, 1.6) | p<0.001 | 1102.6 (1071.4, 1133.8) | p<0.001 |
| 65-74 years | 45,191 | 22.9% | 3,006,666 | 7.4% | 3191.7 (3162.3, 3221.2) | 1.7 (1.7, 1.7) | p<0.001 | 1285.8 (1255.2, 1316.3) | p<0.001 |
| 75-84 years | 40,765 | 20.7% | 1,329,955 | 3.3% | 6509.0 (6445.8, 6572.2) | 1.4 (1.3, 1.4) | p<0.001 | 1670.6 (1604.9, 1736.4) | p<0.001 |
| 85 years and over | 36,070 | 18.3% | 507,505 | 1.2% | 15092.7 (14937.0, 15248.5) | 1.0 (1.0, 1.0) | 0.010 | 191.9 (28.9, 355.0) | 0.011 |
| All ages - crude | 197,170 |  | 40,613,993 |  | 485.5 (483.3, 487.6) | 0.8 (0.8, 0.8) | p<0.001 | -91.9 (-94.3, -89.5) | p<0.001 |
| All ages - age-standardized | 197,170 |  | 40,613,993 |  | 1195.9 (1180.5, 1211.3) | 1.4 (1.4, 1.4) | p<0.001 | 344.2 (328.1, 360.4) | p<0.001 |
| Before 65 (age-standardized) | 75,144 | 38.1% | 35,769,867 | 88.1% | 525.6 (515.8, 535.5) | 1.7 (1.6, 1.7) | p<0.001 | 207.7 (197.3, 218.1) | p<0.001 |
| Before 75 (age standardized) | 120,335 | 61.0% | 38,776,533 | 95.5% | 713.0 (701.8, 724.2) | 2.2 (2.2, 2.3) | p<0.001 | 395.0 (383.3, 406.7) | p<0.001 |
|  |  |  |  |  |  |  |  |  |  |
| **Hispanic** |  |  |  |  |  |  |  |  |  |
| Under 1 year | 1,808 | 1.4% | 1,007,577 | 1.7% | 381.0 (363.5, 398.6) | 1.1 (1.0, 1.1) | 0.003 | 29.6 (8.3, 50.8) | 0.003 |
| 1-4 years | 340 | 0.3% | 4,164,396 | 7.2% | 17.3 (15.5, 19.2) | 1.0 (0.9, 1.1) | 0.561 | -0.2 (-2.4, 2.1) | 0.439 |
| 5-14 years | 506 | 0.4% | 10,535,155 | 18.2% | 10.2 (9.3, 11.1) | 0.9 (0.8, 1.0) | 0.031 | -1.1 (-2.2, 0.0) | 0.029 |
| 15-24 years | 3,178 | 2.5% | 9,814,256 | 17.0% | 68.8 (66.4, 71.2) | 1.1 (1.1, 1.1) | p<0.001 | 6.0 (3.1, 8.8) | p<0.001 |
| 25-34 years | 5,138 | 4.0% | 9,429,166 | 16.3% | 115.7 (112.5, 118.9) | 0.8 (0.8, 0.8) | p<0.001 | -24.8 (-28.6, -21.0) | p<0.001 |
| 35-44 years | 7,279 | 5.7% | 8,587,112 | 14.9% | 180.0 (175.9, 184.1) | 0.8 (0.8, 0.8) | p<0.001 | -43.1 (-48.1, -38.1) | p<0.001 |
| 45-54 years | 11,774 | 9.1% | 7,025,565 | 12.2% | 355.9 (349.5, 362.3) | 0.9 (0.9, 0.9) | p<0.001 | -39.1 (-46.4, -31.8) | p<0.001 |
| 55-64 years | 19,739 | 15.3% | 2,749,799 | 4.8% | 1524.4 (1503.1, 1545.6) | 0.9 (0.9, 0.9) | p<0.001 | -229.7 (-253.0, -206.3) | p<0.001 |
| 65-74 years | 23,853 | 18.5% | 2,682,684 | 4.6% | 1888.1 (1864.2, 1912.1) | 1.0 (1.0, 1.0) | 0.916 | -17.8 (-43.2, 7.5) | 0.084 |
| 75-84 years | 26,308 | 20.4% | 1,236,374 | 2.1% | 4518.6 (4464.0, 4573.2) | 0.9 (0.9, 0.9) | p<0.001 | -319.8 (-377.3, -262.3) | p<0.001 |
| 85 years and over | 28,837 | 22.4% | 499,028 | 0.9% | 12271.2 (12129.6, 12412.8) | 0.8 (0.8, 0.8) | p<0.001 | -2629.6 (-2779.2, -2480.0) | p<0.001 |
| All ages - crude | 128,760 |  | 57,731,112 |  | 223.0 (221.8, 224.3) | 0.4 (0.4, 0.4) | p<0.001 | -354.3 (-355.9, -352.7) | p<0.001 |
| All ages - age-standardized | 128,760 |  | 57,731,112 |  | 760.8 (748.5, 773.1) | 0.9 (0.9, 0.9) | p<0.001 | -90.9 (-104.1, -77.7) | p<0.001 |
| Before 65 (age-standardized) | 49,762 | 38.6% | 53,313,026 | 92.3% | 278.4 (271.7, 285.0) | 0.9 (0.9, 0.9) | p<0.001 | -39.6 (-47.1, -32.2) | p<0.001 |
| Before 75 (age standardized) | 73,615 | 57.2% | 55,995,710 | 97.0% | 391.5 (383.5, 399.5) | 1.2 (1.2, 1.3) | p<0.001 | 73.5 (64.9, 82.2) | p<0.001 |
|  |  |  |  |  |  |  |  |  |  |
| **Non-Hispanic American Indian or Alaskan Native*** |  |  |  |  |  |  |  |  |  |
| Under 1 year | 74 | 0.8% | 38,260 | 1.5% | 410.7 (317.1, 504.3) | 1.2 (0.9, 1.5) | 0.093 | 59.3 (-35.1, 153.6) | 0.109 |
| 1-4 years | 29 | 0.3% | 156,473 | 6.0% | 39.4 (25.0, 53.7) | 2.2 (1.6, 3.3) | p<0.001 | 21.8 (7.5, 36.2) | 0.001 |
| 5-14 years | 31 | 0.3% | 409,393 | 15.8% | 16.1 (10.4, 21.7) | 1.4 (1.0, 2.0) | 0.025 | 4.8 (-0.9, 10.5) | 0.049 |
| 15-24 years | 237 | 2.5% | 419,255 | 16.2% | 120.0 (104.8, 135.3) | 1.9 (1.7, 2.2) | p<0.001 | 57.2 (41.9, 72.6) | p<0.001 |
| 25-34 years | 629 | 6.7% | 418,797 | 16.2% | 318.9 (294.0, 343.9) | 2.3 (2.1, 2.5) | p<0.001 | 178.4 (153.4, 203.4) | p<0.001 |
| 35-44 years | 735 | 7.9% | 333,378 | 12.9% | 468.2 (434.3, 502.0) | 2.1 (1.9, 2.3) | p<0.001 | 245.1 (211.1, 279.0) | p<0.001 |
| 45-54 years | 1,081 | 11.6% | 326,384 | 12.6% | 703.3 (661.4, 745.3) | 1.8 (1.7, 1.9) | p<0.001 | 308.3 (266.3, 350.4) | p<0.001 |
| 55-64 years | 1,760 | 18.8% | 174,263 | 6.7% | 2144.7 (2044.5, 2244.9) | 1.2 (1.2, 1.3) | p<0.001 | 390.7 (290.0, 491.4) | p<0.001 |
| 65-74 years | 1,898 | 20.3% | 202,493 | 7.8% | 1990.4 (1900.9, 2080.0) | 1.0 (1.0, 1.1) | 0.030 | 84.4 (-5.5, 174.4) | 0.033 |
| 75-84 years | 1,695 | 18.1% | 85,020 | 3.3% | 4233.6 (4032.1, 4435.2) | 0.9 (0.8, 0.9) | p<0.001 | -604.7 (-807.1, -402.4) | p<0.001 |
| 85 years and over | 1,170 | 12.5% | 28,950 | 1.1% | 8582.2 (8090.4, 9074.0) | 0.6 (0.5, 0.6) | p<0.001 | -6318.6 (-6812.7, -5824.5) | p<0.001 |
| All ages - crude | 9,339 |  | 2,592,666 |  | 360.2 (352.9, 367.5) | 0.6 (0.6, 0.6) | p<0.001 | -217.1 (-224.5, -209.8) | p<0.001 |
| All ages - age-standardized | 9,339 |  | 2,592,666 |  | 882.5 (830.1, 935.0) | 1.0 (1.0, 1.1) | 0.121 | 30.8 (-21.8, 83.5) | p<0.001 |
| Before 65 (age-standardized) | 4,576 | 49.0% | 2,276,203 | 87.8% | 490.1 (453.0, 527.2) | 1.5 (1.4, 1.7) | p<0.001 | 172.1 (134.8, 209.4) | p<0.001 |
| Before 75 (age standardized) | 6,474 | 69.3% | 2,478,696 | 95.6% | 595.5 (555.3, 635.8) | 1.9 (1.7, 2.0) | p<0.001 | 277.6 (237.1, 318.0) | p<0.001 |
|  |  |  |  |  |  |  |  |  |  |
| **Non-Hispanic Asian Pacific Islander** | |  |  |  |  |  |  |  |  |
| Under 1 year | 260 | 0.6% | 216,177 | 1.1% | 255.4 (224.4, 286.4) | 0.7 (0.6, 0.8) | p<0.001 | -96.1 (-129.3, -62.8) | p<0.001 |
| 1-4 years | 67 | 0.2% | 949,886 | 4.9% | 15.0 (11.4, 18.6) | 0.9 (0.7, 1.1) | 0.111 | -2.5 (-6.4, 1.3) | 0.097 |
| 5-14 years | 92 | 0.2% | 2,429,718 | 12.5% | 8.0 (6.4, 9.7) | 0.7 (0.6, 0.9) | 0.001 | -3.2 (-5.0, -1.5) | p<0.001 |
| 15-24 years | 380 | 0.9% | 2,692,199 | 13.8% | 30.0 (27.0, 33.0) | 0.5 (0.4, 0.5) | p<0.001 | -32.8 (-36.2, -29.5) | p<0.001 |
| 25-34 years | 737 | 1.7% | 3,534,255 | 18.1% | 44.3 (41.1, 47.5) | 0.3 (0.3, 0.3) | p<0.001 | -96.3 (-100.1, -92.4) | p<0.001 |
| 35-44 years | 1,271 | 2.9% | 3,233,519 | 16.6% | 83.5 (78.9, 88.1) | 0.4 (0.3, 0.4) | p<0.001 | -139.6 (-145.0, -134.3) | p<0.001 |
| 45-54 years | 2,555 | 5.8% | 2,759,529 | 14.2% | 196.6 (189.0, 204.2) | 0.5 (0.5, 0.5) | p<0.001 | -198.4 (-206.8, -190.0) | p<0.001 |
| 55-64 years | 5,060 | 11.5% | 1,174,022 | 6.0% | 915.2 (890.0, 940.5) | 0.5 (0.5, 0.5) | p<0.001 | -838.8 (-865.8, -811.8) | p<0.001 |
| 65-74 years | 8,119 | 18.5% | 1,508,767 | 7.7% | 1142.7 (1117.9, 1167.6) | 0.6 (0.6, 0.6) | p<0.001 | -763.3 (-789.4, -737.1) | p<0.001 |
| 75-84 years | 10,618 | 24.2% | 708,822 | 3.6% | 3181.0 (3120.5, 3241.5) | 0.7 (0.6, 0.7) | p<0.001 | -1657.3 (-1720.5, -1594.2) | p<0.001 |
| 85 years and over | 14,662 | 33.5% | 285,572 | 1.5% | 10902.8 (10726.4, 11079.3) | 0.7 (0.7, 0.7) | p<0.001 | -3998.0 (-4180.9, -3815.0) | p<0.001 |
| All ages - crude | 43,821 |  | 19,492,466 |  | 224.8 (222.7, 226.9) | 0.4 (0.4, 0.4) | p<0.001 | -352.5 (-354.9, -350.2) | p<0.001 |
| All ages - age-standardized | 43,821 |  | 19,492,466 |  | 522.8 (508.5, 537.1) | 0.6 (0.6, 0.6) | p<0.001 | -328.9 (-344.0, -313.8) | p<0.001 |
| Before 65 (age-standardized) | 10,422 | 23.8% | 16,989,305 | 87.2% | 155.2 (147.4, 163.1) | 0.5 (0.5, 0.5) | p<0.001 | -162.7 (-171.3, -154.2) | p<0.001 |
| Before 75 (age standardized) | 18,541 | 42.3% | 18,498,072 | 94.9% | 224.6 (215.6, 233.7) | 0.7 (0.7, 0.7) | p<0.001 | -93.3 (-103.0, -83.6) | p<0.001 |

*Caution is required regarding the NHAIAN data, which have well-known problem with accuracy. See References 19-21.
