# Supplementary material for: Correction: Variation in racial/ethnic disparities in COVID-19 mortality by age in the United States: A cross-sectional study
Source: PLoS Med. 2021 Feb 4;18(2):e1003541. doi: 10.1371/journal.pmed.1003541 (PMC7861409; doi:10.1371/journal.pmed.1003541)
Supplement: S2 Table — (DOCX) [file pmed.1003541.s001.docx]

Table S2: Years of Potential Life Lost (YPLL) and age-standardized YPLL rates per 100,000 due to all cause mortality using age 65 and age 75 as cutoffs, with age-standardized YPLL ratios compared to Non-Hispanic whites as of July 22, 2020, United States

|  | Non-Hispanic White | Non-Hispanic Black | Hispanic or Latino | Non-Hispanic American Indian or Alaska Native | Non-Hispanic Asian or Pacific Islander |
| --- | --- | --- | --- | --- | --- |
| **All-cause mortality** |  |  |  |  |  |
| YPLL65 | 3207474  (3189470, 3225479) | 1260058  (1247870, 1272245) | 945646  (934772, 956520) | 84346  (81241, 87451) | 164279  (159991, 168567) |
| YPLL65 difference (compared to Non-Hispanic White) | 0 (reference) | -1947417  (-1969159, -1925675) | -2261828 (-2282862, -2240795) | -3123128 (-3141399, -3104858) | -3043196 (-3061704, -3024687) |
| Age-standardized YPLL65 rate per 100,000 | 1880.5 (1849.0, 1912.0) | 3209.0 (3125.2, 3292.9) | 1683.7 (1634.4, 1732.9) | 3368.7 (3030.0, 3707.4) | 883.8 (817.7, 949.9) |
| Age-standardized YPLL65 rate ratio (compared to Non-Hispanic White) | 1.00 (reference) | 1.7 (1.7, 1.7) | 0.9 (0.9, 0.9) | 1.8 (1.8, 1.8) | 0.5 (0.5, 0.5) |
| YPLL75 | 6526104  (6500062, 6552147) | 2237452  (2220763, 2254142) | 1562531  (1547960, 1577102) | 139596  (135346, 143846) | 309094  (303112, 315076) |
| YPLL75 difference (compared to Non-Hispanic-White) | 0 (reference) | -4288652  (-4319583, -4257721) | -4963574 (-4993415, -4933732) | -6386508 (-6412895, -6360122) | -6217010 (-6243731, -6190290) |
| Age-standardized YPLL75 rate per 100,000 | 3485.0 (3440.8, 3529.3) | 5867.8 (5747.0, 5988.5) | 3122.4 (3048.0, 3196.8) | 5694.4 (5211.2, 6177.6) | 1700.1 (1605.6, 1794.7) |
| Age-standardized YPLL75 rate ratio (compared to Non-Hispanic White) | 1.00 (reference) | 1.7 (1.7, 1.7) | 0.9 (0.9, 0.9) | 1.6 (1.6, 1.7) | 0.5 (0.5, 0.5) |
